# Stability and Fitness Impact of the Visually Discernible Rosea1 Marker in the *Tobacco etch virus* Genome

**DOI:** 10.3390/v5092153

**Published:** 2013-09-09

**Authors:** Eszter Majer, José-Antonio Daròs, Mark P. Zwart

**Affiliations:** Instituto de Biología Molecular y Celular de Plantas, Consejo Superior de Investigaciones Científicas-Universidad Politécnica de Valencia, Ingeniero Fausto Elio s/n, 46022 València, Spain; E-Mails: esma@ibmcp.upv.es (E.M.); jadaros@ibmcp.upv.es (J.-A.D.)

**Keywords:** Rosea1, green fluorescent protein, *Tobacco etch virus*, potyvirus, *Nicotiana tabacum*, stability, genomic deletions, competitive fitness, evolution

## Abstract

*Antirrhinum majus* Rosea1 (Ros1) is an MYB-related transcription factor that induces anthocyanin biosynthesis in plant tissues, and has been shown to be suitable for visual tracking of virus infection in plants. However, activation of anthocyanin biosynthesis has far reaching effects on plant physiology and could consequently have negative effects on viral replication. Therefore, viruses carrying the Ros1 marker might have a low fitness and consequently rapidly lose the marker. To compare the stability of the Ros1 marker, we generated *Tobacco etch virus* (TEV) based constructs containing either Ros1 or the enhanced green fluorescent protein (eGFP) between the NIb and CP cistrons (TEV-Ros1 and TEV-eGFP, respectively). We measured the within-host competitive fitness of both viruses by direct competitions with a common competitor during infection of *Nicotiana tabacum*. The fitness of TEV-Ros1 was significantly lower than that of TEV-eGFP, and both recombinant viruses had a significantly lower fitness than the wild-type virus. Nevertheless, after seven weeks of infection in *N. tabacum*, similar levels of marker gene instability where found for both viruses. Despite lower fitness of the marked virus, Ros1 is therefore a viable alternative marker for tracking viral infection in plants.

## 1. Introduction

Viruses carrying markers have been widely used to study plant-virus infection dynamics, at spatial scales ranging from individual cells to whole plants [1,2,3,4,5,6,7,8,9]. Markers used range from unique sequences that must be detected by PCR-based amplification [7], to those coding for enzymes catalyzing reactions whose products can be detected after processing the tissue [3,10], or to those coding for fluorescent proteins that can be directly detected in infected tissue with the proper instrumentation [1,2]. Recently, a new system that allows tracking infection dynamics throughout the plant with the unaided eye has been developed [11]. This system is based on viral expression of the *Antirrhinum majus* (snapdragon) Rosea1 (Ros1) transcription factor, which induces biosynthesis of colored anthocyanins in infected tissues. A wide range of markers for tracking virus infection is therefore available. The strengths and weaknesses of these markers have, however, often not been documented in detail, with the exception of key attributes such as detection sensitivity and ease of use. To enable rational choices with respect to marker use in research and industry, we here consider potential weaknesses of the Ros1 marker. A comparison in terms of the effects on viral fitness and stability is made with the enhanced green fluorescent protein (eGFP) [1], one of the most commonly used markers to track plant-virus infection at present.

*A. majus* Ros1 is a 25.7-kDa (660 bp at the cDNA level, coding for 220 amino acids) R2R3 MYB transcription factor [12]. It is a component of a triad that also includes a basic helix-loop-helix and a WD40 repeat transcription factors. This triad cooperatively activates transcription of a series of genes involved in anthocyanin biosynthesis in plant tissues [13]. In terms of size, Ros1 is similar to most fluorescent reporter proteins. Whereas fluorescent proteins are considered rather inert, Ros1 is a transcription factor that induces changes in the host gene expression profile. Moreover, anthocyanin accumulation is induced in many plants as a response to biotic and abiotic stresses. Given these reasons for suspecting that Ros1 expression may affect viral infection, we considered two key attributes of the Ros1 marker inserted in the genome of a typical plant RNA virus, *Tobacco etch virus* (TEV; genus *Potyvirus*, family *Potyviridae*): the effects on within-host competitive fitness and on marker stability. Within-host competitive fitness is a measure of the ability of a virus to directly compete with conspecific viruses infecting the same host. This fitness measure therefore indicates to what extent the expression of a marker protein affects the virus capacity to replicate and move. Moreover, when compared to the within-host fitness of the wild-type virus, it will give an indication of the strength of selection for those virus variants that lose the marker gene by means of recombination. However, the actual stability of the virus will also depend on the mutation supply (*i.e.*, the frequency of recombination and the specific recombinants generated) and virus-host interactions. Experimental measures of marker stability are therefore also relevant. In order to assess stability, we therefore analyzed the integrity of the marker sequence after seven weeks of infection in a single plant. These conditions were chosen because it has been shown that deletions in a marker gene are most likely to occur during long passages [14]. Finally, for the sake of completeness we also considered whether the Ros1-marked viruses could be used to quantify primary infection foci.

## 2. Results and Discussion

### 2.1. Virus Constructs

**Figure 1:**
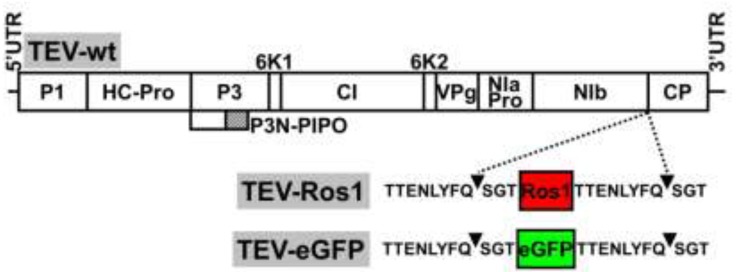
Schemes of wild-type TEV (TEV-wt) and recombinant TEV-Ros1 and TEV-eGFP. Lines represent the viral 5' and 3' untranslated regions (5'UTR and 3'UTR) and boxes represent the viral cistrons P1, HC-Pro, P3, P3N-PIPO, 6K1, CI, 6K2, VPg, NIaPro, NIb and CP, as indicated. The red and green boxes represent Ros1 and eGFP markers as indicated. Sequences of NIaPro cleavage sites flanking the markers in TEV-Ros1 and TEV-eGFP are given. Arrowheads point the exact cleavage position of the protease.

Our aim was to compare some of the properties of the recently developed Ros1 marker with the more classical eGFP as reporters of plant virus infection. We therefore constructed a TEV recombinant clone with an eGFP cDNA inserted between NIb and CP cistrons: TEV-eGFP (Figure 1 and Supplemental Figure 1). This new recombinant clone was absolutely parallel in sequence to our previously described TEV-Ros1 (NIb/CP) [11] (referred to here as TEV-Ros1, for simplicity) except for the sequence of the reporter marker (Figure 1 and Supplemental Figure 1, see Electronic Supplementary Information). In both TEV-Ros1 and TEV-eGFP, the Ros1 and eGFP markers are released from the viral polyprotein by the activity of viral NIa proteinase on properly engineered cleavage sites flanking both markers. The sequences flanking both markers were exactly the same (Supplemental Figure 1). After proteolytic cleavage, both markers are released with three (SGT) and eight (TTENLYFQ) additional amino acids at the amino and carboxyl termini, respectively. These additional peptides result from the engineered NIb/marker and marker/CP cleavage sites (Figure 1).

### 2.2. Quantification of Primary Infection Foci

**Figure 2:**
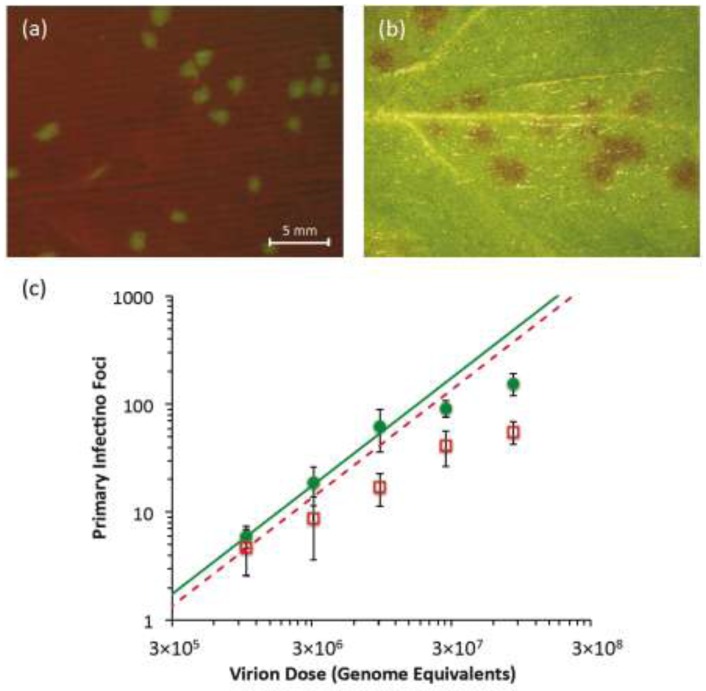
Primary infection foci of TEV-eGFP and TEV-Ros1. In (**a**), fluorescent stereomicroscope image of primary infection foci of TEV-eGFP at 3 dpi (dose = 3.09 × 10^6^ genome equivalents). In (**b**), visible stereomicroscope image of primary infection foci of TEV-Ros1 at 6 dpi (dose = 9.26 × 10^6^ genome equivalents). In panel (**c**), plot of the relationship between dose (abscissae) and the number of foci (ordinate) for TEV-eGFP (green circles) and TEV-Ros1 (red open squares). Error bars denote the standard deviation. To help interpret the data, we used the data from the lowest dose to calculate an infection probability (Section 3.4), and used this value to plot the predicted dose-foci relationship (green line for TEV-eGFP, dotted red line for TEV-Ros1). Note that for the IAH model, the slope of this line is always the same, irrespective of the infection probability.

*Nicotiana tabacum* L. cv. Xanthi plants were initially agroinoculated with TEV-Ros1 and TEV-eGFP. Symptomatic tissues were collected at 7 dpi and aliquoted to more easily produce infectious extracts of both virus variants. When new *N. tabacum* plants were mechanically inoculated with these infectious extracts, individual primary infection foci were detected in the inoculated leaves. In the case of TEV‑eGFP, infection foci were fluorescent and they were detected starting from 3 dpi using a fluorescent stereomicroscope (Figure 2a). In the case of TEV-Ros1, red-colored primary infection foci were detected using a stereomicroscope under visible light conditions, although they were only visible at 5 dpi and were not as clearly defined as TEV-eGFP foci (Figure 2b). When the dose *vs.* foci relationship was plotted (Figure 2c), the number of observed foci appears to start saturating immediately at the lowest dose for TEV-Ros1. For TEV-eGFP, saturation only occurs around 100 foci, whereas the response appears to be linear for lower doses. We then fitted the independent-action hypothesis (IAH) and dependent action (DA) infection models to the full data set using maximum-likelihood methods (Section 3.4). The DA model was better supported for both viruses, as shown by model selection using the Akaike Information Criterion (AIC) (Table 1). This result confirms that the data for both viruses deviate appreciably from the linear dose-foci relationship predicted by the IAH model. On the other hand, if only the lowest three doses were considered, the IAH model was better supported for TEV-eGFP than TEV-Ros1 (Table 1). This result suggests that the dose-foci relationship is only linear for TEV-eGFP at low doses (*i.e.*, doses which result in <100 foci).

**Table 1:** Infection models for dose-foci relationship. Model fitting and selection results are given for the dose-foci relationship (Figure 2c) of TEV-eGFP and TEV-Ros1. Data indicates whether the full data set was used (all doses), or only the partial data set was used (low doses), which comprises only the lowest three doses. Model indicates whether the independent action hypothesis (IAH) or dependent action (DA) model was fitted. Estimated model parameters are the probability of infection per virion (*ρ*) and, for the DA model, a constant that determines the strength of dose-dependent effects on *ρ* (*κ*). For a detailed description of the models see Section 3.4. NLL is negative likelihood, a measure of model fit. AIC is Akaike Information Criterion, ΔAIC is the difference in AIC between the best-fitting model and other models, and AW is the Akaike Weight, the probability that the data provide most support to the model in question. For the full data sets of both viruses, the DA model is better supported than the IAH model. For the low-dose data, the IAH model is better supported for the TEV-eGFP data, indicating a linear response. On the other hand, for TEV-Ros1, the DA model is better supported even when only these data are considered.

Virus	Data	Model	Parameter estimates	NLL	AIC	ΔAIC	AW
TEV-eGFP	All doses	IAH	*ρ* = 2.63 × 10^−6^	302.719	607.438	299.944	0
		DA	*ρ* = 2.95 × 10^−3^ ; *κ* = 0.60	151.747	397.494	-	1
	Low doses	IAH	*ρ* = 6.46 × 10^−6^	61.456	124.912	-	0.601
		DA	*ρ* = 1.86 × 10^−6^ ; *κ* = 1.08	60.865	125.730	0.818	0.399
TEV-Ros1	All doses	IAH	*ρ* = 1.02 × 10^−6^	139.048	280.097	129.090	0
		DA	*ρ* = 2.69 × 10^−3^; *κ* = 0.55	73.504	151.007	-	1
	Low doses	IAH	*ρ* = 2.29 × 10^−6^	39.012	80.024	10.508	0.005
		DA	*ρ* = 1.32 × 10^−3^; *κ* = 0.59	32.758	69.516	-	0.995

The number of primary infection foci renders an estimate of virus effective population size (*N_e_*) for virus infection of a plant [8], because *N_e_* can be approximated as the smallest census size for rapidly expanding and contracting population [15]. Here we found that TEV-Ros1 did not perform as well as TEV-eGFP for the quantification of primary infection foci. Infection foci were only visible starting from 5 dpi, at which point they were too diffuse to quantify well at high virus doses. Moreover, the dose-foci relationship appears to saturate much more quickly for TEV-Ros1 than for TEV-eGFP. In contrast to eGFP, the Ros1 marker may therefore be unsuitable for accurate quantification of primary infection foci. However, the appearance of primary infection foci may depend on exact inoculation and growing conditions, and on the virus in which the marker is inserted. For example, during inoculation of larger leaves in older plants, individual primary infection foci were more easily discriminated [16]. Moreover, the marker may still be useful in experiments where a semi‑quantitative assessment of the primary infection foci is needed. The dose-foci relationship has been reported to saturate for TEV‑eGFP at high doses [5]. Unlike TEV-Ros1, TEV-eGFP foci can be readily quantified when the number of foci <100 [8,9]. Rejection of the IAH model at high TEV-eGFP doses could be due to real deviations from the IAH model, such as a limited number of infectious sites in the inoculated leaf [17]. On the other hand, it might simply be difficult to distinguish foci when high doses are used and there are a large number of foci.

### 2.3. Within-Host Competitive Fitness of TEV-Ros1 and TEV-eGFP

**Figure 3:**
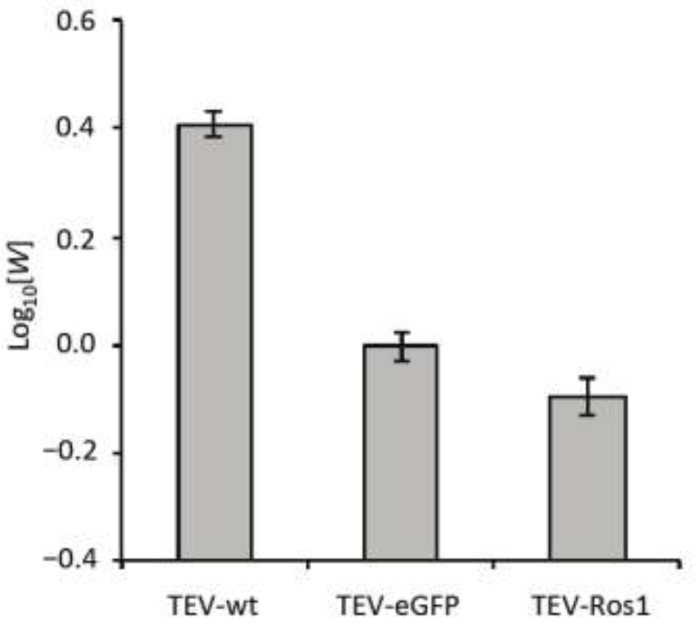
Competitive within-host fitness of TEV-wt TEV-eGFP and TEV-Ros1. Plot of the log_10_-transformed within-host competitive fitness (*W*) for wild-type TEV (TEV-wt) and its two derived viruses (TEV-eGFP and TEV-Ros1) carrying markers, as measured by direct competitions with TEV-mCherry. Error bars indicate the standard deviation.

We quantified the within-host competitive fitness (*W*; see Section 3.5) of wild-type TEV (TEV-wt) [11], TEV-Ros1 and TEV-eGFP in *N. tabacum* using TEV-mCherry [8] (a similar virus variant carrying the fluorescent protein mCherry in0 between the P1 and HC-Pro cistrons) as a common competitor (Figure 3).

Overall, there were highly significant differences between the viruses in fitness (ANOVA: *F*_2,12_ = 432.381, *P* < 0.001). TEV-wt had a significantly higher within-host fitness than the TEV-eGFP (independent samples t-test with Holm-Bonferroni correction were used as a *post hoc* test: *t* = 25.213, 8 d.f., *P* < 0.001), whereas TEV-Ros1 had a significantly lower fitness than TEV-eGFP (*t* = −4.795, 8 d.f., *P* = 0.001). Therefore, even though TEV-Ros1 has a slightly shorter marker sequence than TEV‑eGFP, its fitness was significantly lower. The difference between TEV-eGFP and TEV-Ros1 is, however, small compared to differences between these two viruses and TEV-wt.

The within-host competitive fitness data have two ramifications that should be given consideration. First, infection dynamics of both marked viruses will probably not be exactly the same as the wild-type virus, given that there is a fitness cost associated to the marker. Second, the difference in fitness between TEV-Ros1 and a TEV-Ros1-derived deletion mutant can in principle be larger than for TEV‑eGFP, although this will depend on the exact deletion variant. Therefore, ceteris paribus a TEV-Ros1 population could show substantial losses of the marker before a TEV-eGFP population.

### 2.4. Marker Instability in TEV-Ros1 and TEV-eGFP

**Figure 4:**
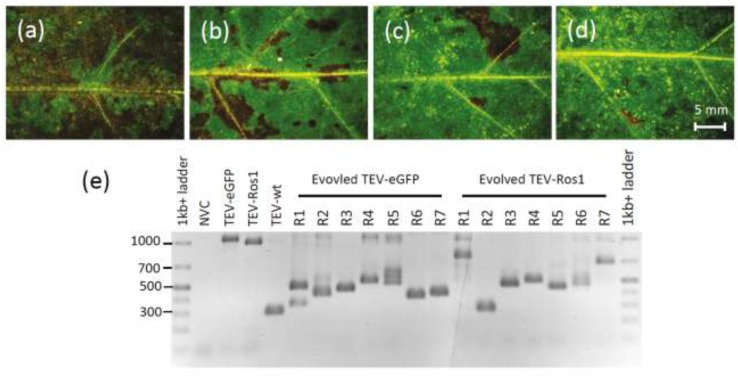
(**a–d**) Leaves 10, 15, 20 and 25, respectively, of a plant infected with TEV-Ros1 after seven weeks of infection. Although all the tissues shown are fully symptomatic, anthocyanin expression appears to decrease, being almost entirely lost in leaf 25. RT-qPCR confirmed the loss of the marker gene in this particular plant. (**e**) An agarose gel containing RT-PCR products with primers flanking the marker gene is shown. The GeneRuler 1 kb+ DNA Ladder (Thermo Scientific) was used and NVC is a non-virus control (mock‑inoculated plant). Note that in most of the 7-week-infected plants there are PCR products smaller than the ancestral virus visible, indicating the occurrence of deletions in the marker gene.

After seven weeks of infection in *N. tabacum*, we assessed the integrity of the marker gene for TEV-Ros1 and TEV-eGFP, using RT-PCR with primers flanking the marker gene on RNA extracted from the 30th leaf (Section 3.6). Visual examination revealed that deletions of the marker gene had probably occurred in TEV-Ros1-infected plants (Figure 4a–d). Note that direct visual examination of TEV-eGFP-infected plants is not informative. RT-PCR showed that most plants had accumulated deletions at this point for both marked viruses, and in some plants the ancestral viruses could no longer be detected (Figure 4e). We then classified the plants based on the presence of the ancestral virus and did not find a significant difference between the TEV-Ros1 and TEV-eGFP (Table 2). We also performed a second analysis comparing the evolved populations of TEV-Ros1 and TEV-eGFP, which considers both the length and relative intensity of PCR amplicons by means of a weighted mean (Section 3.6). With this procedure we also found no significant differences between the two viruses (Mann-Whitney *U*-test: *P* = 0.865). Our data therefore suggest that the stability of TEV-Ros1 and TEV-eGFP are similar, despite the minor difference in within-host competitive fitness.

**Figure 5:**
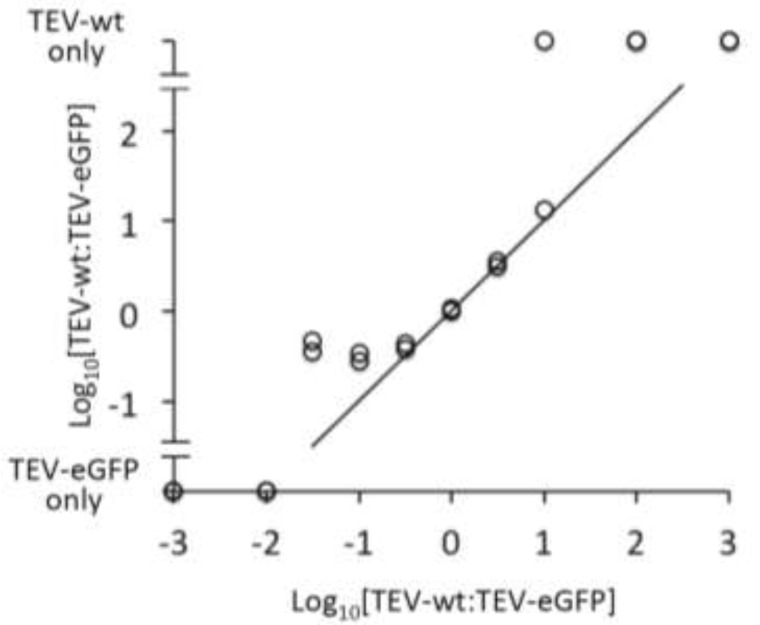
The results of a control experiment to determine whether smaller PCR amplicons were selectively amplified are given. The abscissa is the log_10_-transformed input ratio, whilst the ordinate is the log_10_-transformed output ratio. Note that samples containing only TEV-wt or TEV-eGFP are also depicted on the ordinate. Circles represent the data, and the 1:1 relationship between input and output is also given for reference.

During a PCR, smaller products may be amplified with a greater efficiency than larger products [18]. Selective amplification of smaller products could in turn bias our comparison of the weighted means of PCR amplicons towards smaller products, lowering the sensitivity of this test. We therefore performed a control experiment to ensure the quantification of the PCR amplicons did not strongly bias the results (Section 3.6). In essence, we compared known input ratios to the output ratio generated by the assay. The results (Figure 5) show that our PCR assay is not very sensitive: the rare variant was never detected with ratios TEV-wt:TEV-eGFP ≤1:100 or ≥100:1. However, for a range from 3:1 to 1:10 the input and output of the control were very similar. For the experimental RT-PCR stability data, the vast majority of samples had a high frequency of deletion variants, and low frequencies of the ancestral marker virus. These conditions therefore coincide well with the range in which the assay appears to work best, supporting the use of our test for differences in marker stability using weighted means. Note that we did not perform a correction for the size of PCR amplicons (*i.e.*, output is the raw ratio of the signal of the two amplicons on gel). Therefore, under the conditions used the assay appears to work well because the higher efficiency of PCR amplification for smaller products [18] is counterbalanced by their lower intensity in an agarose gel stained with ethidium bromide.

**Table 2:** Marker instability for TEV-Ros1 and TEV-eGFP. After seven weeks of infection in *N. tabacum*, RT-PCR was used to detect deletions in the marker gene in individual plants. We then categorized the evolved populations as either containing the ancestral virus (*i.e.*, having a PCR amplicon corresponding to the ancestral virus) or not. There was not a significant difference between the two viruses (*χ*^2^ test: *χ*^2^ = 1.414, 1 d.f., *P* = 0.234). Note that for both viruses, in two plants we detected only ancestral virus and no deletion mutants (and hence here they are classified as having the ancestral virus present), an insignificant difference (Binomial test: *P* = 1).

Virus	Detection of ancestral virus by RT-PCR	
	Present	Absent
TEV-Ros1	25 (67.6%)	12 (32.4%)
TEV-eGFP	16 (53.3%)	14 (46.7%)

Two tests of marker instability were performed: a *χ*^2^ test for the presence of the ancestral virus and a second procedure developed here, a Mann-Whitney U-test on the mean relative amplicon length for the genomic region containing the marker. The latter procedure may in principle be useful for detecting more subtle differences between evolved virus populations than simply the presence or absence of the ancestral virus (*i.e*., the *χ*^2^ test). For example, the amplicon-length-based procedure might still detect differences if (i) the frequency of the ancestral virus tends to be higher in the evolved populations of one virus type, but (ii) the number of lineages in which deletions of the marker gene has occurred is the same for both virus types. In practice, however, for both procedures we did not find a difference in instability between the two viruses, and there is therefore not yet any evidence that the amplicon-length-based procedure has greater power for detecting differences in instability.

After seven weeks of infection plants infected with TEV-Ros1 were significantly shorter than those infected with TEV-eGFP (Mann-Whitney *U*-test: *P* < 0.001), suggesting that Ros1 expression is adversely affecting plant growth. When the Ros1 maker was lost during virus expansion, it appeared as if plants grew faster in those regions of the plant in which virus expressing Ros1 were no longer present. However, this effect was not statistically significant for either virus; those plants in which we sampled only viruses that did not express the marker gene in the 30th leaf were not significantly larger than plants in which marker genes were detected (Mann-Whitney *U*-test; TEV-Ros1: *P* = 0.355, TEV‑eGFP: *P* = 0.181).

The *N. tabacum* genome includes the *An2* gene, coding for an MYB transcription factor and a homolog of *A. majus* Ros1 [19]. During infection RNA silencing mechanisms might, therefore, potentially interfere either with the expression of the endogenous *An2* or the virally expressed Ros1. However, our experimental observations suggest that interference does not occur. One explanation for the lack of RNA silencing in infected tissues is the activity of TEV HC-Pro, a strong suppressor of RNA silencing. Nonetheless, subtle alterations in expression of the endogenous *An2* may contribute to the larger phenotypic effects induced by Ros1, in contrast to eGFP.

Finally, the distribution of mutational effects on viral fitness might be different for the eGFP and Ros1 markers. Single-nucleotide mutations in the eGFP marker are unlikely to have an effect on viral fitness, because fitness is independent of fluorophore activity. On the other hand, mutations in Ros1 leading to a loss of anthocyanin-biosynthesis induction may be beneficial for viral fitness. Nevertheless, TEV has a relatively low mutation rate [20] and single-nucleotide mutations have been observed to accumulate slowly during experimental evolution [21]. These observations suggest that in the vast majority of cases the marker protein will be inactivated by genomic deletions rather than single‑nucleotide mutations.

## 3. Experimental Section

### 3.1. Generation of Recombinant TEV-Ros1 and TEV-GFP Viruses

Plasmid pGTEVa [11] contains the TEV cDNA (GenBank accession number DQ986288), including two silent mutations (G273A and A1119G) to eliminate internal Eco31I restriction sites, under the control of 35S promoter and terminator of *Cauliflower mosaic virus*. Plasmids pGTEV-Ros1 and pGTEV-eGFP derived directly from pGTEVa. In these plasmids the cDNAs of Ros1 (DQ275529) or eGFP (AAB08060) were inserted in between the TEV nuclear inclusion b (NIb) and coat protein (CP) cistrons by standard techniques based on the use of the type IIS restriction enzyme Eco31I (Thermo Scientific) and T4 DNA ligase (Thermo Scientific) [22]. pGTEV-Ros1 was previously referred to as pGTEV-Ros1(NIb/CP) [11]. The complete sequences of the TEV recombinant clones expressed from these plasmids are in the Supplemental Figure 1.

### 3.2. Plant Inoculation

Four-week-old tobacco plants cultivated in a growth chamber under a photoperiod of 16 h day and 8 h night at 24°C were agroinoculated in the third true leaf with cultures of *Agrobacterium tumefaciens* C58C1 containing the pCLEAN-S48 helper plasmid [23] and plasmids pGTEVa, pGTEV-Ros1 or pGTEV-eGFP. Prior to agroinoculation, bacteria were grown in liquid culture, adjusted to an OD (600 nm) of 0.5 and induced for 2 h at 28 °C with 150 µM acetosyringone in 10 mM MES-NaOH, pH 5.6, 10 mM MgCl_2_ [24]. Symptomatic tissue was collected seven days post-inoculation (dpi) and used for subsequent mechanical inoculation.

Infectious extracts were obtained by homogenizing infected tissue with five volumes of inoculation buffer (50 mM potassium phosphate, pH 7.0, 3% polyethylene glycol 6000). *N. tabacum* plants (as described above) were mechanically inoculated by gently rubbing 5 μL virus extract and a 2-µL drop of 10% Carborundum in inoculation buffer onto the third true leaf.

### 3.3. RT-qPCR to Determine Load of Virus Genome Equivalents

Total RNA was extracted from approximately 100 mg of virus-infected tissue using the Invitrap Spin Plant RNA Mini Kit (Stratec Molecular). The Primescript RT-PCR Kit II (Takara) and a Step One Plus thermocycler (Applied Biosystems) were then used to perform a SYBR Green I-based one-step RT-qPCR, with TEV-specific CP primers: forward primer 5'-TTGGTCTTGATGGCAACGTG-3' and reverse primer 5'-TGTGCCGTTCAGTGTCTTCCT-3'. Absolute quantification using the standard curve method was performed, using the Step One v 2.2 software [25] to analyze the data. For generating a standard curve, full-length TEV-mCherry RNA was transcribed *in vitro* from the pTEV-mCherry plasmid [8]. This RNA was then purified, RNA concentration was quantified by Nanodrop, and RNA integrity was checked by agarose gel. TEV genome copy numbers in the purified RNA were calculated from RNA concentration. Five-fold serial dilutions of RNA ranging from 10^8^ to 3.2 × 10^4^ virus genome copies per µL, in a final concentration of 50 ng/µL RNA from a non-virus control plant, were used as the template for the standard curve [26]. Using the RT-qPCR data, we then calculated the number of TEV genome equivalents per mg of tissue in the viral stocks, including corrections for the amount of tissue used to extract RNA and the dilution of RNA prior to RT-qPCR.

### 3.4. Quantification and Analysis of Primary Infection Foci

Extracts from tissues infected with TEV-eGFP and TEV-Ros1 were diluted with inoculation buffer to a concentration of 1.67 × 10^7^ genome equivalents per μL. Three-fold serial dilutions were then made in inoculation buffer. Five 4-week-old *N. tabacum* plants were then mechanically inoculated on the third true leaf with each virus dilution or inoculation buffer for non-virus controls. Plants were then kept in a growth chamber at 24 °C with 16 h light. For quantifying TEV-eGFP primary infection foci, a Leica MZ16F stereomicroscope with 0.5× objective and GFP2 filters (Leica) was used [8]. TEV‑Ros1 primary infection foci were also observed under the stereomicroscope with visible light.

To analyze the data, we fit two simple infection models to the data. The independent action hypothesis (IAH) model [8,27] assumes that the number of primary infection foci (*f*) is the product of the probability of infection per virion (*ρ*) and virion dose (*d*). This model results in a linear relationship between dose and the number of foci. The dependent action (DA) model [28] assumes that there are dose-dependent effects on the probability of infection, such that 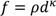, where *κ* is constant that determines the strength of dose-dependent effects. When *κ* > 1 there are synergistic effects, when *κ* < 1 there are antagonistic effects, and when *κ* = 1 the DA model collapses to the IAH model without dose‑dependent effects on the probability of infection per virion. Both models were fitted to the data using a maximum-likelihood approach. Given that the distribution of primary infection foci for a given dose follows approximately a Poisson distribution for low and intermediate doses [8,9], the likelihood of any observed number of foci *k* is 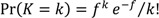. The negative log likelihood was then minimized by grid searches using R 2.14 [29], rendering model parameter estimates and allowing for model selection using the AIC.

### 3.5. Measurement of Within-Host Competitive Fitness

Competition experiments were performed following an approach proposed elsewhere [30]. RT‑qPCR was used to determine the viral load for TEV-wt, TEV-Ros1, TEV-eGFP and TEV-mCherry, a TEV recombinant clone expressing the mCherry fluorescent marker protein between the P1 and HC‑Pro cistrons [8]. All viruses were normalized to 2.5 × 10^7^ genome equivalents per μL using inoculation buffer. TEV-wt, TEV-Ros1 and TEV-eGFP were then mixed with TEV-mCherry in 3:1 mixtures, and five 4-week-old *N. tabacum* plants were mechanically inoculated with each mixture. Under these conditions, the TEV‑eGFP:TEV-mCherry mixture rendered approximately a 3:1 ratio of primary infection foci at 3 dpi.

Infected tissue was harvested 7 dpi, and RNA was extracted, again using the Invitrap Spin Plant RNA Mini Kit. We then performed RT-qPCR with the CP primers (Section 3.3), which were capable of detecting all viruses used in the experiment, to determine the overall viral load. A second, separate one-step RT-qPCR for the mCherry sequence using specific primers (forward primer 5'-CGGCGAGTTCATCTACAAGG-3', and reverse primer 5'-TGGTCTTCTTCTGCATTACGG-3') was then performed. For both primer sets, purified and quantified pTEV-mCherry RNA could again be used for generating a standard curve (Section 3.3), because it contains both template sequences. The ratio *R* of the virus being tested (*i.e.*, TEV-wt, TEV-Ros1, TEV-eGFP) to the common competitor (*i.e.*, TEV-mCherry) is then 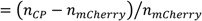, where *n_CP_* and *n_mCherry_* are the copy numbers of CP and mCherry cistrons, respectively, as measured using the RT-qPCR assay. We then use the approach of [26] to measure the replicative advantage (*W*) such that 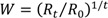. Here *t* is the time in days, *R_t_* is the virus ratio at the end of the experiment, and *R_0_* is the virus ratio at the start of the experiment. Note that we used a 3:1 mixture of the two viruses in the inoculum, instead of a 1:1 mixture, for methodological reasons. *R* can only be accurately measured when *n_mCherry_* levels are considerably lower (*i.e.*, one-half fold) than *n_CP_* levels. We anticipated that the within-host competitive fitness of TEV-Ros1 might be low, and starting with a 3:1 ratio ensures *n_mCherry_* levels remain lower than *n_CP_* levels.

### 3.6. Analysis of Marker Instability

*N. tabacum* plants were mechanically inoculated with inoculation buffer (mock-inoculated control), TEV-wt, TEV-eGFP and TEV-Ros1, and infection was allowed to proceed for 7 weeks. We then measured plant height and counted the number of fully formed leaves. We collected the 30th leaf, or if plants did not have 30 leaves yet, the highest fully formed leaf. RNA was extracted from each leaf using the Invitrap Spin Plant RNA Mini Kit. RT was performed using M-MuLV (Thermo Scientific) and the primer 5'-CGCACTACATAGGAGAATTAG-3'. A Taq DNA polymerase (Roche Applied Science) based PCR was then performed using the forward primer 5'-TACGATATTCCAACGACTG-3' and reverse primer 5'-GCAAACTGCTCATGTGTGG-3'. These primers flank the site of Ros1/eGFP insertion in the TEV genome. PCR products were then resolved by electrophoresis on 1% agarose gels, and ImageJ [31] software was used to quantify the relative frequency of bands within a sample, if multiple bands were present. In order to compare the stability of the insert in the two viruses, a 2 × 2 contingency table contrasting the two viruses and whether, in the evolved virus populations, the ancestral virus (*i.e*., viruses with an intact marker gene) was present or absent was constructed. A *χ*^2^ test was then performed to test for differences between the two viruses. Note that a contingency table also including ‘only ancestral virus’ category was not used because this latter category would have less than 5 observations for both viruses, precluding the use of a *χ*^2^ test.

In an attempt to increase the statistical power of the analysis of marker gene stability, we also performed a second test. The proportion of the respective marker gene present (*α*) was first estimated from the length of the PCR product, rounded off to the nearest 100 bp. We use the proportion of marker instead of simply the length of the PCR product, since eGFP is slightly longer than Ros1 and a significant difference could otherwise simply reflect the length of the marker gene in the ancestral viruses. The ImageJ-derived estimates of the relative intensity of a particular PCR amplicon on the gel within a sample (*β*) were then used to generate a weighted mean (*μ*) for the *z* bands in each individual sample (*i.e.*, 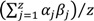). A Mann-Whitney U-test was then performed to test for significant differences in *μ* values for the two viruses.

This second test of stability assumes that the PCR products are at least a qualitative representation of the different virus variants in the sample. In order to test this assumption, we made mixtures of the purified and Nanodrop-quantified plasmids p15TV3 (to represent TEV-wt) and p15TV3-eGFP (to represent TEV-eGFP). These plasmids contain inserts with the approximately last third (from restriction site Eco81I) of the TEV genome. p15TV3-eGFP includes the eGFP marker in a similar situation as the TEV-eGFP recombinant clone. The following molar mixtures (p15TV3:p15TV3-eGFP) were made: 1,000:1, 100:1, 10:1, 1:1, 1:10, 1:100 and 1:1,000, whilst the total amount of DNA was the same for each reaction (10^10^ plasmids). We then performed the PCR and quantification of amplicons on agarose gel as described above. Based on the results obtained, we performed a second experiment in which the molar mixtures 30:1, 3:1 and 1:3 were used. Two independent replicates of both series of mixtures were performed.

## 4. Conclusions

We found that TEV-Ros1 had a significantly lower within-host competitive fitness than TEV-eGFP —although the difference in fitness was small—and both viruses clearly had a lower fitness than TEV‑wt. However, our data indicate that there is not a significant difference in marker loss in TEV‑eGFP and TEV-Ros1. This result suggests that the dynamics of marker loss in a virus population will probably also depend on the mutation supply and virus-host interactions. Therefore, a relatively small difference in within-host competitive fitness may not lead to appreciable differences in marker stability. Previously, it was shown that the Ros1 marker is stable in the TEV genome after six plant-to-plant passages of 9 days [11]. A relation between marker loss and passage time has already been shown for the GUS marker [14] and the eGFP marker [30] in the TEV genome. The results presented here clearly show that the effects of different markers on viral fitness depend on more than simply the length of the marker, since carriage of the shorter Ros1 marker results in a significantly lower fitness than the longer eGFP. This observation suggests that inferring a relationship between genome size and fitness from viruses carrying different markers [32] is unwarranted; different markers can result in different virus-host interactions.

## Supplementary Files

Supplementary File 1 Supplementary Materials (PDF, 33 KB)
