# Supplementary material for: Stability and Fitness Impact of the Visually Discernible Rosea1 Marker in the Tobacco etch virus Genome
Source: Viruses. 2013 Sep 9;5(9):2153–68. doi: 10.3390/v5092153 (PMC3798895; doi:10.3390/v5092153)
Supplement: Supplementary File 1 — Supplementary Materials (PDF, 33 KB) [file viruses-05-02153-s001.pdf]

Supplementary material

Supplemental Fig. 1. Complete sequences of recombinant viral clones TEV-Ros1 and TEV-eGFP. Sequences corresponding to Ros1 and eGFP are highlighted on red and green backgrounds, respectively. Additional sequences fused to the Ros1 and eGFP cDNA 5’ and 3’ termini, corresponding to engineered NIaPro cleavage sites, are on yellow background. TEV cistron borders are underlined. TEV silent mutations G273A and A1119G are shown in red.

>TEV-Ros1

GAAAATAACAAATCTCAACACAACATATACAAAACAAACGAATCTCAAGCAATCAAGCATTCTACTTCTATTGCAGCAATTT
AAATCATTTCTTTTAAAGCAAAAGCAATTTTCTGAAAATTTTCACCATTTACGAACGATAGCCATGGCACTCATCTTTGGCA
CAGTCAACGCTAACATCCTGAAGGAAGTGTTCGGTGGAGCTCGTATGGCTTGCGTTACCAGCGCACATATGGCTGGAGCGAA
TGGAAGCATTTTGAAGAAGGCAGAAGAAACCTCTCGTGCAATCATGCACAAACCAGTGATCTTCGGAGAAGACTACATTACC
GAGGCAGACTTGCCTTACACACCACTCCATTTAGAGGTCGATGCTGAAATGGAGCGGATGTATTATCTTGGTCGTCGCGCGC
TCACCCATGGCAAGAGACGCAAAGTTTCTGTGAATAACAAGAGGAACAGGAGAAGGAAAGTGGCCAAAACGTACGTGGGGCG
TGATTCCATTGTTGAGAAGATTGTAGTGCCCCACACCGAGAGAAAGGTTGATACCACAGCAGCAGTGGAAGACATTTGCAAT
GAAGCTACCACTCAACTTGTGCATAATAGTATGCCAAAGCGTAAGAAGCAGAAAAACTTCTTGCCCGCCACTTCACTAAGTA
ACGTGTATGCCCAAACTTGGAGCATAGTGCGCAAACGCCATATGCAGGTGGAGATCATTAGCAAGAAGAGCGTCCGAGCGAG
GGTCAAGAGATTTGAGGGCTCGGTGCAATTGTTCGCAAGTGTGCGTCACATGTATGGCGAGAGGAAAAGGGTGGACTTACGT
ATTGACAACTGGCAGCAAGAGACACTTCTAGACCTTGCTAAAAGATTTAAGAATGAGAGAGTGGATCAATCGAAGCTCACTT
TTGGTTCAAGTGGCCTAGTTTTGAGGCAAGGCTCGTACGGACCTGCGCATTGGTATCGACATGGTATGTTCATTGTACGCGG
TCGGTCGGATGGGATGTTGGTGGATGCTCGTGCGAAGGTAACGTTCGCTGTTTGTCACTCAATGACACATTATAGCGACAAA
TCAATCTCTGAGGCATTCTTCATACCATACTCTAAGAAATTCTTGGAGTTGAGGCCAGATGGAATCTCCCATGAGTGTACAA
GAGGAGTATCAGTTGAGCGGTGCGGTGAGGTGGCTGCAATCCTGACACAAGCACTTTCACCGTGTGGTAAGATCACATGCAA
ACGTTGCATGGTTGAAACACCTGACATTGTTGAGGGTGAGTCGGGAGACAGTGTCACCAACCAAGGTAAGCTCCTAGCAATG
CTGAAAGAACAGTATCCAGATTTCCCAATGGCCGAGAAACTACTCACAAGGTTTTTGCAACAGAAATCACTAGTAAATACAA
ATTTGACAGCCTGCGTGAGCGTCAAACAACTCATTGGTGACCGCAAACAAGCTCCATTCACACACGTACTGGCTGTCAGCGA
AATTCTGTTTAAAGGCAATAAACTAACAGGGGCCGATCTCGAAGAGGCAAGCACACATATGCTTGAAATAGCAAGGTTCTTG
AACAATCGCACTGAAAATATGCGCATTGGCCACCTTGGTTCTTTCAGAAATAAAATCTCATCGAAGGCCCATGTGAATAACG
CACTCATGTGTGATAATCAACTTGATCAGAATGGGAATTTTATTTGGGGACTAAGGGGTGCACACGCAAAGAGGTTTCTTAA
AGGATTTTTCACTGAGATTGACCCAAATGAAGGATACGATAAGTATGTTATCAGGAAACATATCAGGGGTAGCAGAAAGCTA
GCAATTGGCAATTTGATAATGTCAACTGACTTCCAGACGCTCAGGCAACAAATTCAAGGCGAAACTATTGAGCGTAAAGAAA
TTGGGAATCACTGCATTTCAATGCGGAATGGTAATTACGTGTACCCATGTTGTTGTGTTACTCTTGAAGATGGTAAGGCTCA
ATATTCGGATCTAAAGCATCCAACGAAGAGACATCTGGTCATTGGCAACTCTGGCGATTCAAAGTACCTAGACCTTCCAGTT
CTCAATGAAGAGAAAATGTATATAGCTAATGAAGGTTATTGCTACATGAACATTTTCTTTGCTCTACTAGTGAATGTCAAGG
AAGAGGATGCAAAGGACTTCACCAAGTTTATAAGGGACACAATTGTTCCAAAGCTTGGAGCGTGGCCAACAATGCAAGATGT
TGCAACTGCATGCTACTTACTTTCCATTCTTTACCCAGATGTCCTGAGTGCTGAATTACCCAGAATTTTGGTTGATCATGAC
AACAAAACAATGCATGTTTTGGATTCGTATGGGTCTAGAACGACAGGATACCACATGTTGAAAATGAACACAACATCCCAGC
TAATTGAATTCGTTCATTCAGGTTTGGAATCCGAAATGAAAACTTACAATGTTGGAGGGATGAACCGAGATATGGTCACACA
AGGTGCAATTGAGATGTTGATCAAGTCCATATACAAACCACATCTCATGAAGCAGTTACTTGAGGAGGAGCCATACATAATT
GTCCTGGCAATAGTCTCCCCTTCAATTTTAATTGCCATGTACAACTCTGGAACTTTTGAGCAGGCGTTACAAATGTGGTTGC
CAAATACAATGAGGTTAGCTAACCTCGCTGCCATCTTGTCAGCCTTGGCGCAAAAGTTAACTTTGGCAGACTTGTTCGTCCA
GCAGCGTAATTTGATTAATGAGTATGCGCAGGTAATTTTGGACAATCTGATTGACGGTGTCAGGGTTAACCATTCGCTATCC
CTAGCAATGGAAATTGTTACTATTAAGCTGGCCACCCAAGAGATGGACATGGCGTTGAGGGAAGGTGGCTATGCTGTGACCT
CTGAAAAGGTGCATGAAATGTTGGAAAAAAACTATGTAAAGGCTTTGAAGGATGCATGGGACGAATTAACTTGGTTGGAAAA
ATTCTCCGCAATCAGGCATTCAAGAAAGCTCTTGAAATTTGGGCGAAAGCCTTTAATCATGAAAAACACCGTAGATTGCGGC
GGACATATAGACTTGTCTGTGAAATCGCTTTTCAAGTTCCACTTGGAACTCCTGAAGGGAACCATCTCAAGAGCCGTAAATG
GTGGTGCAAGAAAGGTAAGAGTAGCGAAGAATGCCATGACAAAAGGGGTTTTTCTCAAAATCTACAGCATGCTTCCTGACGT
CTACAAGTTTATCACAGTCTCGAGTGTCCTTTCCTTGTTGTTGACATTCTTATTTCAAATTGACTGCATGATAAGGGCACAC
CGAGAGGCGAAGGTTGCTGCACAGTTGCAGAAAGAGAGCGAGTGGGACAATATCATCAATAGAACTTTCCAGTATTCTAAGC
TTGAAAATCCTATTGGCTATCGCTCTACAGCGGAGGAAAGACTCCAATCAGAACACCCCGAGGCTTTCGAGTACTACAAGTT
TTGCATTGGAAAGGAAGACCTCGTTGAACAGGCAAAACAACCGGAGATAGCATACTTTGAAAAGATTATAGCTTTCATCACA
CTTGTATTAATGGCTTTTGACGCTGAGCGGAGTGATGGAGTGTTCAAGATACTCAATAAGTTCAAAGGAATACTGAGCTCAA
CGGAGAGGGAGATCATCTACACGCAGAGTTTGGATGATTACGTTACAACCTTTGATGACAATATGACAATCAACCTCGAGTT
GAATATGGATGAACTCCACAAGACGAGCCTTCCTGGAGTCACTTTTAAGCAATGGTGGAACAACCAAATCAGCCGAGGCAAC
GTGAAGCCACATTATAGAACTGAGGGGCACTTCATGGAGTTTACCAGAGATACTGCGGCATCGGTTGCCAGCGAGATATCAC
ACTCACCCGCAAGAGATTTTCTTGTGAGAGGTGCTGTTGGATCTGGAAAATCCACAGGACTTCCATACCATTTATCAAAGAG
AGGGAGAGTGTTAATGCTTGAGCCTACCAGACCACTCACAGATAACGTGCACAAGCAACTGAGAAGTGAACCATTTAACTGC
TTCCCAACTTTGAGGATGAGAGGGAAGTCAACTTTTGGGTCATCACCGATTACAGTCATGACTAGTGGATTCGCTTTACACC

ATTTTGCACGAAACATAGCTGAGGTAAAAACATACGATTTTGTCATAATTGATGAATGTCATGTGAATGATGCTTCTGCTAT
AGCGTTTAGGAATCTACTGTTTGAACATGAATTTGAAGGAAAAGTCCTCAAAGTGTCAGCCACACCACCAGGTAGAGAAGTT
GAATTCACAACTCAGTTTCCCGTGAAACTCAAGATAGAAGAGGCTCTTAGCTTTCAGGAATTTGTAAGTTTACAAGGGACAG
GTGCCAACGCCGATGTGATTAGTTGTGGCGACAACATACTAGTATATGTTGCTAGCTACAATGATGTTGATAGTCTTGGCAA
GCTCCTTGTGCAAAAGGGATACAAAGTGTCGAAGATTGATGGAAGAACAATGAAGAGTGGAGGAACTGAAATAATCACTGAA
GGTACTTCAGTGAAAAAGCATTTCATAGTCGCAACTAATATTATTGAGAATGGTGTAACCATTGACATTGATGTAGTTGTGG
ATTTTGGGACTAAGGTTGTACCAGTTTTGGATGTGGACAATAGAGCGGTGCAGTACAACAAAACTGTGGTGAGTTATGGGGA
GCGCATCCAAAGACTCGGTAGAGTTGGGCGACACAAGGAAGGAGTAGCACTTCGAATTGGCCAAACAAATAAAACACTGGTT
GAAATTCCAGAAATGGTTGCCACTGAAGCTGCCTTTCTATGCTTCATGTACAATTTGCCAGTGACAACACAGAGTGTTTCAA
CCACACTGCTGGAAAATGCCACATTATTACAAGCTAGAACTATGGCACAGTTTGAGCTATCATATTTTTACACAATTAATTT
TGTGCGATTTGATGGTAGTATGCATCCAGTCATACATGACAAGCTGAAGCGCTTTAAGCTACACACTTGTGAGACATTCCTC
AATAAGTTGGCGATCCCAAATAAAGGCTTATCCTCTTGGCTTACGAGTGGAGAGTATAAGCGACTTGGTTACATAGCAGAGG
ATGCTGGCATAAGAATCCCATTCGTGTGCAAAGAAATTCCAGACTCCTTGCATGAGGAAATTTGGCACATTGTAGTCGCCCA
TAAAGGTGACTCGGGTATTGGGAGGCTCACTAGCGTACAGGCAGCAAAGGTTGTTTATACTCTGCAAACGGATGTGCACTCA
ATTGCGAGGACTCTAGCATGCATCAATAGACTCATAGCACATGAACAAATGAAGCAGAGTCATTTTGAAGCCGCAACTGGGA
GAGCATTTTCCTTCACAAATTACTCAATACAAAGCATATTTGACACGCTGAAAGCAAATTATGCTACAAAGCATACGAAAGA
AAATATTGCAGTGCTTCAGCAGGCAAAAGATCAATTGCTAGAGTTTTCGAACCTAGCAAAGGATCAAGATGTCACGGGTATC
ATCCAAGACTTCAATCACCTGGAAACTATCTATCTCCAATCAGATAGCGAAGTGGCTAAGCATCTGAAGCTTAAAAGTCACT
GGAATAAAAGCCAAATCACTAGGGACATCATAATAGCTTTGTCTGTGTTAATTGGTGGTGGATGGATGCTTGCAACGTACTT
CAAGGACAAGTTCAATGAACCAGTCTATTTCCAAGGGAAGAAGAATCAGAAGCACAAGCTTAAGATGAGAGAGGCGCGTGGG
GCTAGAGGGCAATATGAGGTTGCAGCGGAGCCAGAGGCGCTAGAACATTACTTTGGAAGCGCATATAATAACAAAGGAAAGC
GCAAGGGCACCACGAGAGGAATGGGTGCAAAGTCTCGGAAATTCATAAACATGTATGGGTTTGATCCAACTGATTTTTCATA
CATTAGGTTTGTGGATCCATTGACAGGTCACACTATTGATGAGTCCACAAACGCACCTATTGATTTAGTGCAGCATGAGTTT
GGAAAGGTTAGAACACGCATGTTAATTGACGATGAGATAGAGCCTCAAAGTCTTAGCACCCACACCACAATCCATGCTTATT
TGGTGAATAGTGGCACGAAGAAAGTTCTTAAGGTTGATTTAACACCACACTCGTCGCTACGTGCGAGTGAGAAATCAACAGC
AATAATGGGATTTCCTGAAAGGGAGAATGAATTGCGTCAAACCGGCATGGCAGTGCCAGTGGCTTATGATCAATTGCCACCA
AAGAGTGAGGACTTGACGTTTGAAGGAGAAAGCTTGTTTAAGGGACCACGTGATTACAACCCGATATCGAGCACCATTTGTC
ACTTGACGAATGAATCTGATGGGCACACAACATCGTTGTATGGTATTGGATTTGGTCCCTTCATCATTACAAACAAGCACTT
GTTTAGAAGAAATAATGGAACACTGTTGGTCCAATCACTACATGGTGTATTCAAGGTCAAGAACACCACGACTTTGCAACAA
CACCTCATTGATGGGAGGGACATGATAATTATTCGCATGCCTAAGGATTTCCCACCATTTCCTCAAAAGCTGAAATTTAGAG
AGCCACAAAGGGAAGAGCGCATATGTCTTGTGACAACCAACTTCCAAACTAAGAGCATGTCTAGCATGGTGTCAGACACTAG
TTGCACATTCCCTTCATCTGATGGCATATTCTGGAAGCATTGGATTCAAACCAAGGATGGGCAGTGTGGCAGTCCATTAGTA
TCAACTAGAGATGGGTTCATTGTTGGTATACACTCAGCATCGAATTTCACCAACACAAACAATTATTTCACAAGCGTGCCGA
AAAACTTCATGGAATTGTTGACAAATCAGGAGGCGCAGCAGTGGGTTAGTGGTTGGCGATTAAATGCTGACTCAGTATTGTG
GGGGGGCCATAAAGTTTTCATGAGCAAACCTGAAGAGCCTTTTCAGCCAGTTAAGGAAGCGACTCAACTCATGAGTGAATTG
GTGTACTCGCAAGGGGAGAAGAGGAAATGGGTCGTGGAAGCACTGTCAGGGAACTTGAGGCCAGTGGCTGAGTGTCCCAGTC
AGTTAGTCACAAAGCATGTGGTTAAAGGAAAGTGTCCCCTCTTTGAGCTCTACTTGCAGTTGAATCCAGAAAAGGAAGCATA
TTTTAAACCGATGATGGGAGCATATAAGCCAAGTCGACTTAATAGAGAGGCGTTCCTCAAGGACATTCTAAAATATGCTAGT
GAAATTGAGATTGGGAATGTGGATTGTGACTTGCTGGAGCTTGCAATAAGCATGCTCATCACAAAGCTCAAGGCGTTAGGAT
TCCCAACTGTGAACTACATCACTGACCCAGAGGAAATTTTTAGTGCATTGAATATGAAAGCAGCTATGGGAGCACTATACAA
AGGCAAGAAGAAAGAAGCTCTCAGCGAGCTCACACTAGATGAGCAGGAGGCAATGCTCAAAGCAAGTTGCCTGCGACTGTAT
ACGGGAAAGCTGGGAATTTGGAATGGCTCATTGAAAGCAGAGTTGCGTCCAATTGAGAAGGTTGAAAACAACAAAACGCGAA
CTTTCACAGCAGCACCAATAGACACTCTTCTTGCTGGTAAAGTTTGCGTGGATGATTTCAACAATCAATTTTATGATCTCAA
CATAAAGGCACCATGGACAGTTGGTATGACTAAGTTTTATCAGGGGTGGAATGAATTGATGGAGGCTTTACCAAGTGGGTGG
GTGTATTGTGACGCTGATGGTTCGCAATTCGACAGTTCCTTGACTCCATTCCTCATTAATGCTGTATTGAAAGTGCGACTTG
CCTTCATGGAGGAATGGGATATTGGTGAGCAAATGCTGCGAAATTTGTACACTGAGATAGTGTATACACCAATCCTCACACC
GGATGGTACTATCATTAAGAAGCATAAAGGCAACAATAGCGGGCAACCTTCAACAGTGGTGGACAACACACTCATGGTCATT
ATTGCAATGTTATACACATGTGAGAAGTGTGGAATCAACAAGGAAGAGATTGTGTATTACGTCAATGGCGATGACCTATTGA
TTGCCATTCACCCAGATAAAGCTGAGAGGTTGAGTGGATTCAAAGAATCTTTCGGAGAGTTGGGCCTGAAATATGAATTTGA
CTGCACCACCAGGGACAAGACACAGTTGTGGTTCATGTCACACAGGGCTTTGGAGAGGGATGGCATGTATATACCAAAGCTA
GAAGAAGAAAGGATTGTTTCTATTTTGGAATGGGACAGATCCAAAGAGCCGTCACATAGGCTTGAAGCCATCTGTGCATCAA
TGATCGAAGCATGGGGTTATGACAAGCTGGTTGAAGAAATCCGCAATTTCTATGCATGGGTTTTGGAACAAGCGCCGTATTC
ACAGCTTGCAGAAGAAGGAAAGGCGCCATATCTGGCTGAGACTGCGCTTAAGTTTTTGTACACATCTCAGCACGGAACAAAC
TCTGAGATAGAAGAGTATTTAAAAGTGTTGTATGATTACGATATTCCAACGACTGAGAATCTTTATTTTCAGTCAGGTACAA
TGGAAAAGAATTGTCGTGGAGTGAGAAAAGGTACTTGGACCAAAGAAGAAGACACTCTCTTGAGGCAATGTATAGAAGAGTA
TGGTGAAGGGAAATGGCATCAAGTTCCACACAGAGCAGGGTTGAACCGGTGTAGGAAGAGTTGCAGGCTGAGGTGGTTGAAT
TATCTGAGGCCAAATATCAAAAGAGGTCGGTTTTCGAGAGATGAAGTGGACCTAATTGTGAGGCTTCATAAGCTGTTGGGTA
ACAAATGGTCGCTGATTGCTGGTAGAATTCCTGGAAGGACAGCTAATGACGTGAAGAACTTTTGGAATACTCATGTGGGGAA
GAATTTAGGCGAGGATGGAGAACGATGCCGGAAAAATGTTATGAACACAAAAACCATTAAGCTGACTAATATCGTAAGACCC
CGAGCTCGGACCTTCACCGGATTGCACGTTACTTGGCCGAGAGAAGTCGGAAAAACCGATGAATTTTCAAATGTCCGGTTAA
CAACTGATGAGATTCCAGATTGTGAGAAGCAAACGCAATTTTACAATGATGTTGCGTCGCCACAAGATGAAGTTGAAGACTG
CATTCAGTGGTGGAGTAAGTTGCTAGAAACAACGGAGGATGGGGAATTAGGAAACCTATTCGAGGAGGCCCAACAAATTGGA
AATACTACAGAGAACCTCTACTTTCAAAGTGGCACTGTGGGTGCTGGTGTTGACGCTGGTAAGAAGAAAGATCAAAAGGATG

ATAAAGTCGCTGAGCAGGCTTCAAAGGATAGGGATGTTAATGCTGGAACTTCAGGAACATTCTCAGTTCCACGAATAAATGC
TATGGCCACAAAACTTCAATATCCAAGGATGAGGGGAGAGGTGGTTGTAAACTTGAATCACCTTTTAGGATACAAGCCACAG
CAAATTGATTTGTCAAATGCTCGAGCCACACATGAGCAGTTTGCCGCGTGGCATCAGGCAGTGATGACAGCCTATGGAGTGA
ATGAAGAGCAAATGAAAATATTGCTAAATGGATTTATGGTGTGGTGCATAGAAAATGGGACTTCCCCAAATTTGAACGGAAC
TTGGGTTATGATGGATGGTGAGGAGCAAGTTTCATACCCGCTGAAACCAATGGTTGAAAACGCGCAGCCAACACTGAGGCAA
ATTATGACACACTTCAGTGACCTGGCTGAAGCGTATATTGAGATGAGGAATAGGGAGCGACCATACATGCCTAGGTATGGTC
TACAGAGAAACATTACAGACATGAGTTTGTCACGCTATGCGTTCGACTTCTATGAGCTAACTTCAAAAACACCTGTTAGAGC
GAGGGAGGCGCATATGCAAATGAAAGCTGCTGCAGTACGAAACAGTGGAACTAGGTTATTTGGTCTTGATGGCAACGTGGGT
ACTGCAGAGGAAGACACTGAACGGCACACAGCGCACGATGTGAACCGTAACATGCACACACTATTAGGGGTCCGCCAGTGAT
AGTTTCTGCGTGTCTTTGCTTTCCGCTTTTAAGCTTATTGTAATATATATGAATAGCTATTCACAGTGGGACTTGGTCTTGT
GTTGAATGGTATCTTATATGTTTTAATATGTCTTATTAGTCTCATTACTTAGGCGAACGACAAAGTGAGGTCACCTCGGTCT
AATTCTCCTATGTAGTGCGAGAAAAAAAAAAAAAAAAAAAAAAAAAAAAAAAAAAAAAAAAAAAAAAAA

>TEV-eGFP
GAAAATAACAAATCTCAACACAACATATACAAAACAAACGAATCTCAAGCAATCAAGCATTCTACTTCTATTGCAGCAATTT
AAATCATTTCTTTTAAAGCAAAAGCAATTTTCTGAAAATTTTCACCATTTACGAACGATAGCCATGGCACTCATCTTTGGCA
CAGTCAACGCTAACATCCTGAAGGAAGTGTTCGGTGGAGCTCGTATGGCTTGCGTTACCAGCGCACATATGGCTGGAGCGAA
TGGAAGCATTTTGAAGAAGGCAGAAGAAACCTCTCGTGCAATCATGCACAAACCAGTGATCTTCGGAGAAGACTACATTACC
GAGGCAGACTTGCCTTACACACCACTCCATTTAGAGGTCGATGCTGAAATGGAGCGGATGTATTATCTTGGTCGTCGCGCGC
TCACCCATGGCAAGAGACGCAAAGTTTCTGTGAATAACAAGAGGAACAGGAGAAGGAAAGTGGCCAAAACGTACGTGGGGCG
TGATTCCATTGTTGAGAAGATTGTAGTGCCCCACACCGAGAGAAAGGTTGATACCACAGCAGCAGTGGAAGACATTTGCAAT
GAAGCTACCACTCAACTTGTGCATAATAGTATGCCAAAGCGTAAGAAGCAGAAAAACTTCTTGCCCGCCACTTCACTAAGTA
ACGTGTATGCCCAAACTTGGAGCATAGTGCGCAAACGCCATATGCAGGTGGAGATCATTAGCAAGAAGAGCGTCCGAGCGAG
GGTCAAGAGATTTGAGGGCTCGGTGCAATTGTTCGCAAGTGTGCGTCACATGTATGGCGAGAGGAAAAGGGTGGACTTACGT
ATTGACAACTGGCAGCAAGAGACACTTCTAGACCTTGCTAAAAGATTTAAGAATGAGAGAGTGGATCAATCGAAGCTCACTT
TTGGTTCAAGTGGCCTAGTTTTGAGGCAAGGCTCGTACGGACCTGCGCATTGGTATCGACATGGTATGTTCATTGTACGCGG
TCGGTCGGATGGGATGTTGGTGGATGCTCGTGCGAAGGTAACGTTCGCTGTTTGTCACTCAATGACACATTATAGCGACAAA
TCAATCTCTGAGGCATTCTTCATACCATACTCTAAGAAATTCTTGGAGTTGAGGCCAGATGGAATCTCCCATGAGTGTACAA
GAGGAGTATCAGTTGAGCGGTGCGGTGAGGTGGCTGCAATCCTGACACAAGCACTTTCACCGTGTGGTAAGATCACATGCAA
ACGTTGCATGGTTGAAACACCTGACATTGTTGAGGGTGAGTCGGGAGACAGTGTCACCAACCAAGGTAAGCTCCTAGCAATG
CTGAAAGAACAGTATCCAGATTTCCCAATGGCCGAGAAACTACTCACAAGGTTTTTGCAACAGAAATCACTAGTAAATACAA
ATTTGACAGCCTGCGTGAGCGTCAAACAACTCATTGGTGACCGCAAACAAGCTCCATTCACACACGTACTGGCTGTCAGCGA
AATTCTGTTTAAAGGCAATAAACTAACAGGGGCCGATCTCGAAGAGGCAAGCACACATATGCTTGAAATAGCAAGGTTCTTG
AACAATCGCACTGAAAATATGCGCATTGGCCACCTTGGTTCTTTCAGAAATAAAATCTCATCGAAGGCCCATGTGAATAACG
CACTCATGTGTGATAATCAACTTGATCAGAATGGGAATTTTATTTGGGGACTAAGGGGTGCACACGCAAAGAGGTTTCTTAA
AGGATTTTTCACTGAGATTGACCCAAATGAAGGATACGATAAGTATGTTATCAGGAAACATATCAGGGGTAGCAGAAAGCTA
GCAATTGGCAATTTGATAATGTCAACTGACTTCCAGACGCTCAGGCAACAAATTCAAGGCGAAACTATTGAGCGTAAAGAAA
TTGGGAATCACTGCATTTCAATGCGGAATGGTAATTACGTGTACCCATGTTGTTGTGTTACTCTTGAAGATGGTAAGGCTCA
ATATTCGGATCTAAAGCATCCAACGAAGAGACATCTGGTCATTGGCAACTCTGGCGATTCAAAGTACCTAGACCTTCCAGTT
CTCAATGAAGAGAAAATGTATATAGCTAATGAAGGTTATTGCTACATGAACATTTTCTTTGCTCTACTAGTGAATGTCAAGG
AAGAGGATGCAAAGGACTTCACCAAGTTTATAAGGGACACAATTGTTCCAAAGCTTGGAGCGTGGCCAACAATGCAAGATGT
TGCAACTGCATGCTACTTACTTTCCATTCTTTACCCAGATGTCCTGAGTGCTGAATTACCCAGAATTTTGGTTGATCATGAC
AACAAAACAATGCATGTTTTGGATTCGTATGGGTCTAGAACGACAGGATACCACATGTTGAAAATGAACACAACATCCCAGC
TAATTGAATTCGTTCATTCAGGTTTGGAATCCGAAATGAAAACTTACAATGTTGGAGGGATGAACCGAGATATGGTCACACA
AGGTGCAATTGAGATGTTGATCAAGTCCATATACAAACCACATCTCATGAAGCAGTTACTTGAGGAGGAGCCATACATAATT
GTCCTGGCAATAGTCTCCCCTTCAATTTTAATTGCCATGTACAACTCTGGAACTTTTGAGCAGGCGTTACAAATGTGGTTGC
CAAATACAATGAGGTTAGCTAACCTCGCTGCCATCTTGTCAGCCTTGGCGCAAAAGTTAACTTTGGCAGACTTGTTCGTCCA
GCAGCGTAATTTGATTAATGAGTATGCGCAGGTAATTTTGGACAATCTGATTGACGGTGTCAGGGTTAACCATTCGCTATCC
CTAGCAATGGAAATTGTTACTATTAAGCTGGCCACCCAAGAGATGGACATGGCGTTGAGGGAAGGTGGCTATGCTGTGACCT
CTGAAAAGGTGCATGAAATGTTGGAAAAAAACTATGTAAAGGCTTTGAAGGATGCATGGGACGAATTAACTTGGTTGGAAAA
ATTCTCCGCAATCAGGCATTCAAGAAAGCTCTTGAAATTTGGGCGAAAGCCTTTAATCATGAAAAACACCGTAGATTGCGGC
GGACATATAGACTTGTCTGTGAAATCGCTTTTCAAGTTCCACTTGGAACTCCTGAAGGGAACCATCTCAAGAGCCGTAAATG
GTGGTGCAAGAAAGGTAAGAGTAGCGAAGAATGCCATGACAAAAGGGGTTTTTCTCAAAATCTACAGCATGCTTCCTGACGT
CTACAAGTTTATCACAGTCTCGAGTGTCCTTTCCTTGTTGTTGACATTCTTATTTCAAATTGACTGCATGATAAGGGCACAC
CGAGAGGCGAAGGTTGCTGCACAGTTGCAGAAAGAGAGCGAGTGGGACAATATCATCAATAGAACTTTCCAGTATTCTAAGC
TTGAAAATCCTATTGGCTATCGCTCTACAGCGGAGGAAAGACTCCAATCAGAACACCCCGAGGCTTTCGAGTACTACAAGTT
TTGCATTGGAAAGGAAGACCTCGTTGAACAGGCAAAACAACCGGAGATAGCATACTTTGAAAAGATTATAGCTTTCATCACA
CTTGTATTAATGGCTTTTGACGCTGAGCGGAGTGATGGAGTGTTCAAGATACTCAATAAGTTCAAAGGAATACTGAGCTCAA
CGGAGAGGGAGATCATCTACACGCAGAGTTTGGATGATTACGTTACAACCTTTGATGACAATATGACAATCAACCTCGAGTT
GAATATGGATGAACTCCACAAGACGAGCCTTCCTGGAGTCACTTTTAAGCAATGGTGGAACAACCAAATCAGCCGAGGCAAC
GTGAAGCCACATTATAGAACTGAGGGGCACTTCATGGAGTTTACCAGAGATACTGCGGCATCGGTTGCCAGCGAGATATCAC

ACTCACCCGCAAGAGATTTTCTTGTGAGAGGTGCTGTTGGATCTGGAAAATCCACAGGACTTCCATACCATTTATCAAAGAG
AGGGAGAGTGTTAATGCTTGAGCCTACCAGACCACTCACAGATAACGTGCACAAGCAACTGAGAAGTGAACCATTTAACTGC
TTCCCAACTTTGAGGATGAGAGGGAAGTCAACTTTTGGGTCATCACCGATTACAGTCATGACTAGTGGATTCGCTTTACACC
ATTTTGCACGAAACATAGCTGAGGTAAAAACATACGATTTTGTCATAATTGATGAATGTCATGTGAATGATGCTTCTGCTAT
AGCGTTTAGGAATCTACTGTTTGAACATGAATTTGAAGGAAAAGTCCTCAAAGTGTCAGCCACACCACCAGGTAGAGAAGTT
GAATTCACAACTCAGTTTCCCGTGAAACTCAAGATAGAAGAGGCTCTTAGCTTTCAGGAATTTGTAAGTTTACAAGGGACAG
GTGCCAACGCCGATGTGATTAGTTGTGGCGACAACATACTAGTATATGTTGCTAGCTACAATGATGTTGATAGTCTTGGCAA
GCTCCTTGTGCAAAAGGGATACAAAGTGTCGAAGATTGATGGAAGAACAATGAAGAGTGGAGGAACTGAAATAATCACTGAA
GGTACTTCAGTGAAAAAGCATTTCATAGTCGCAACTAATATTATTGAGAATGGTGTAACCATTGACATTGATGTAGTTGTGG
ATTTTGGGACTAAGGTTGTACCAGTTTTGGATGTGGACAATAGAGCGGTGCAGTACAACAAAACTGTGGTGAGTTATGGGGA
GCGCATCCAAAGACTCGGTAGAGTTGGGCGACACAAGGAAGGAGTAGCACTTCGAATTGGCCAAACAAATAAAACACTGGTT
GAAATTCCAGAAATGGTTGCCACTGAAGCTGCCTTTCTATGCTTCATGTACAATTTGCCAGTGACAACACAGAGTGTTTCAA
CCACACTGCTGGAAAATGCCACATTATTACAAGCTAGAACTATGGCACAGTTTGAGCTATCATATTTTTACACAATTAATTT
TGTGCGATTTGATGGTAGTATGCATCCAGTCATACATGACAAGCTGAAGCGCTTTAAGCTACACACTTGTGAGACATTCCTC
AATAAGTTGGCGATCCCAAATAAAGGCTTATCCTCTTGGCTTACGAGTGGAGAGTATAAGCGACTTGGTTACATAGCAGAGG
ATGCTGGCATAAGAATCCCATTCGTGTGCAAAGAAATTCCAGACTCCTTGCATGAGGAAATTTGGCACATTGTAGTCGCCCA
TAAAGGTGACTCGGGTATTGGGAGGCTCACTAGCGTACAGGCAGCAAAGGTTGTTTATACTCTGCAAACGGATGTGCACTCA
ATTGCGAGGACTCTAGCATGCATCAATAGACTCATAGCACATGAACAAATGAAGCAGAGTCATTTTGAAGCCGCAACTGGGA
GAGCATTTTCCTTCACAAATTACTCAATACAAAGCATATTTGACACGCTGAAAGCAAATTATGCTACAAAGCATACGAAAGA
AAATATTGCAGTGCTTCAGCAGGCAAAAGATCAATTGCTAGAGTTTTCGAACCTAGCAAAGGATCAAGATGTCACGGGTATC
ATCCAAGACTTCAATCACCTGGAAACTATCTATCTCCAATCAGATAGCGAAGTGGCTAAGCATCTGAAGCTTAAAAGTCACT
GGAATAAAAGCCAAATCACTAGGGACATCATAATAGCTTTGTCTGTGTTAATTGGTGGTGGATGGATGCTTGCAACGTACTT
CAAGGACAAGTTCAATGAACCAGTCTATTTCCAAGGGAAGAAGAATCAGAAGCACAAGCTTAAGATGAGAGAGGCGCGTGGG
GCTAGAGGGCAATATGAGGTTGCAGCGGAGCCAGAGGCGCTAGAACATTACTTTGGAAGCGCATATAATAACAAAGGAAAGC
GCAAGGGCACCACGAGAGGAATGGGTGCAAAGTCTCGGAAATTCATAAACATGTATGGGTTTGATCCAACTGATTTTTCATA
CATTAGGTTTGTGGATCCATTGACAGGTCACACTATTGATGAGTCCACAAACGCACCTATTGATTTAGTGCAGCATGAGTTT
GGAAAGGTTAGAACACGCATGTTAATTGACGATGAGATAGAGCCTCAAAGTCTTAGCACCCACACCACAATCCATGCTTATT
TGGTGAATAGTGGCACGAAGAAAGTTCTTAAGGTTGATTTAACACCACACTCGTCGCTACGTGCGAGTGAGAAATCAACAGC
AATAATGGGATTTCCTGAAAGGGAGAATGAATTGCGTCAAACCGGCATGGCAGTGCCAGTGGCTTATGATCAATTGCCACCA
AAGAGTGAGGACTTGACGTTTGAAGGAGAAAGCTTGTTTAAGGGACCACGTGATTACAACCCGATATCGAGCACCATTTGTC
ACTTGACGAATGAATCTGATGGGCACACAACATCGTTGTATGGTATTGGATTTGGTCCCTTCATCATTACAAACAAGCACTT
GTTTAGAAGAAATAATGGAACACTGTTGGTCCAATCACTACATGGTGTATTCAAGGTCAAGAACACCACGACTTTGCAACAA
CACCTCATTGATGGGAGGGACATGATAATTATTCGCATGCCTAAGGATTTCCCACCATTTCCTCAAAAGCTGAAATTTAGAG
AGCCACAAAGGGAAGAGCGCATATGTCTTGTGACAACCAACTTCCAAACTAAGAGCATGTCTAGCATGGTGTCAGACACTAG
TTGCACATTCCCTTCATCTGATGGCATATTCTGGAAGCATTGGATTCAAACCAAGGATGGGCAGTGTGGCAGTCCATTAGTA
TCAACTAGAGATGGGTTCATTGTTGGTATACACTCAGCATCGAATTTCACCAACACAAACAATTATTTCACAAGCGTGCCGA
AAAACTTCATGGAATTGTTGACAAATCAGGAGGCGCAGCAGTGGGTTAGTGGTTGGCGATTAAATGCTGACTCAGTATTGTG
GGGGGGCCATAAAGTTTTCATGAGCAAACCTGAAGAGCCTTTTCAGCCAGTTAAGGAAGCGACTCAACTCATGAGTGAATTG
GTGTACTCGCAAGGGGAGAAGAGGAAATGGGTCGTGGAAGCACTGTCAGGGAACTTGAGGCCAGTGGCTGAGTGTCCCAGTC
AGTTAGTCACAAAGCATGTGGTTAAAGGAAAGTGTCCCCTCTTTGAGCTCTACTTGCAGTTGAATCCAGAAAAGGAAGCATA
TTTTAAACCGATGATGGGAGCATATAAGCCAAGTCGACTTAATAGAGAGGCGTTCCTCAAGGACATTCTAAAATATGCTAGT
GAAATTGAGATTGGGAATGTGGATTGTGACTTGCTGGAGCTTGCAATAAGCATGCTCATCACAAAGCTCAAGGCGTTAGGAT
TCCCAACTGTGAACTACATCACTGACCCAGAGGAAATTTTTAGTGCATTGAATATGAAAGCAGCTATGGGAGCACTATACAA
AGGCAAGAAGAAAGAAGCTCTCAGCGAGCTCACACTAGATGAGCAGGAGGCAATGCTCAAAGCAAGTTGCCTGCGACTGTAT
ACGGGAAAGCTGGGAATTTGGAATGGCTCATTGAAAGCAGAGTTGCGTCCAATTGAGAAGGTTGAAAACAACAAAACGCGAA
CTTTCACAGCAGCACCAATAGACACTCTTCTTGCTGGTAAAGTTTGCGTGGATGATTTCAACAATCAATTTTATGATCTCAA
CATAAAGGCACCATGGACAGTTGGTATGACTAAGTTTTATCAGGGGTGGAATGAATTGATGGAGGCTTTACCAAGTGGGTGG
GTGTATTGTGACGCTGATGGTTCGCAATTCGACAGTTCCTTGACTCCATTCCTCATTAATGCTGTATTGAAAGTGCGACTTG
CCTTCATGGAGGAATGGGATATTGGTGAGCAAATGCTGCGAAATTTGTACACTGAGATAGTGTATACACCAATCCTCACACC
GGATGGTACTATCATTAAGAAGCATAAAGGCAACAATAGCGGGCAACCTTCAACAGTGGTGGACAACACACTCATGGTCATT
ATTGCAATGTTATACACATGTGAGAAGTGTGGAATCAACAAGGAAGAGATTGTGTATTACGTCAATGGCGATGACCTATTGA
TTGCCATTCACCCAGATAAAGCTGAGAGGTTGAGTGGATTCAAAGAATCTTTCGGAGAGTTGGGCCTGAAATATGAATTTGA
CTGCACCACCAGGGACAAGACACAGTTGTGGTTCATGTCACACAGGGCTTTGGAGAGGGATGGCATGTATATACCAAAGCTA
GAAGAAGAAAGGATTGTTTCTATTTTGGAATGGGACAGATCCAAAGAGCCGTCACATAGGCTTGAAGCCATCTGTGCATCAA
TGATCGAAGCATGGGGTTATGACAAGCTGGTTGAAGAAATCCGCAATTTCTATGCATGGGTTTTGGAACAAGCGCCGTATTC
ACAGCTTGCAGAAGAAGGAAAGGCGCCATATCTGGCTGAGACTGCGCTTAAGTTTTTGTACACATCTCAGCACGGAACAAAC
TCTGAGATAGAAGAGTATTTAAAAGTGTTGTATGATTACGATATTCCAACGACTGAGAATCTTTATTTTCAGTCAGGTACAA
TGGTGAGCAAGGGCGAGGAGCTGTTCACCGGGGTGGTGCCCATCCTGGTCGAGCTGGACGGCGACGTAAACGGCCACAAGTT
CAGCGTGTCCGGCGAGGGCGAGGGCGATGCCACCTACGGCAAGCTGACCCTGAAGTTCATCTGCACCACCGGCAAGCTGCCC
GTGCCCTGGCCCACCCTCGTGACCACCCTGACCTACGGCGTGCAGTGCTTCAGCCGCTACCCCGACCACATGAAGCAGCACG
ACTTCTTCAAGTCCGCCATGCCCGAAGGCTACGTCCAGGAGCGCACCATCTTCTTCAAGGACGACGGCAACTACAAGACCCG
CGCCGAGGTGAAGTTCGAGGGCGACACCCTGGTGAACCGCATCGAGCTGAAGGGCATCGACTTCAAGGAGGACGGCAACATC
CTGGGGCACAAGCTGGAGTACAACTACAACAGCCACAACGTCTATATCATGGCCGACAAGCAGAAGAACGGCATCAAGGTGA

ACTTCAAGATCCGCCACAACATCGAGGACGGCAGCGTGCAGCTCGCCGACCACTACCAGCAGAACACCCCCATCGGCGACGG
CCCCGTGCTGCTGCCCGACAACCACTACCTGAGCACCCAGTCCGCCCTGAGCAAAGACCCCAACGAGAAGCGCGATCACATG
GTCCTGCTGGAGTTCGTGACCGCCGCCGGGATCACTCTCGGCATGGACGAGCTGTACAAGACTACAGAGAACCTCTACTTTC
AAAGTGGCACTGTGGGTGCTGGTGTTGACGCTGGTAAGAAGAAAGATCAAAAGGATGATAAAGTCGCTGAGCAGGCTTCAAA
GGATAGGGATGTTAATGCTGGAACTTCAGGAACATTCTCAGTTCCACGAATAAATGCTATGGCCACAAAACTTCAATATCCA
AGGATGAGGGGAGAGGTGGTTGTAAACTTGAATCACCTTTTAGGATACAAGCCACAGCAAATTGATTTGTCAAATGCTCGAG
CCACACATGAGCAGTTTGCCGCGTGGCATCAGGCAGTGATGACAGCCTATGGAGTGAATGAAGAGCAAATGAAAATATTGCT
AAATGGATTTATGGTGTGGTGCATAGAAAATGGGACTTCCCCAAATTTGAACGGAACTTGGGTTATGATGGATGGTGAGGAG
CAAGTTTCATACCCGCTGAAACCAATGGTTGAAAACGCGCAGCCAACACTGAGGCAAATTATGACACACTTCAGTGACCTGG
CTGAAGCGTATATTGAGATGAGGAATAGGGAGCGACCATACATGCCTAGGTATGGTCTACAGAGAAACATTACAGACATGAG
TTTGTCACGCTATGCGTTCGACTTCTATGAGCTAACTTCAAAAACACCTGTTAGAGCGAGGGAGGCGCATATGCAAATGAAA
GCTGCTGCAGTACGAAACAGTGGAACTAGGTTATTTGGTCTTGATGGCAACGTGGGTACTGCAGAGGAAGACACTGAACGGC
ACACAGCGCACGATGTGAACCGTAACATGCACACACTATTAGGGGTCCGCCAGTGATAGTTTCTGCGTGTCTTTGCTTTCCG
CTTTTAAGCTTATTGTAATATATATGAATAGCTATTCACAGTGGGACTTGGTCTTGTGTTGAATGGTATCTTATATGTTTTA
ATATGTCTTATTAGTCTCATTACTTAGGCGAACGACAAAGTGAGGTCACCTCGGTCTAATTCTCCTATGTAGTGCGAGAAAA
AAAAAAAAAAAAAAAAAAAAAAAAAAAAAAAAAAAAAAAAA
